# The relationship between autonomous motivation and autonomy support in medical students’ academic achievement

**DOI:** 10.5116/ijme.5843.1097

**Published:** 2016-12-29

**Authors:** Rose Feri, Diantha Soemantri, Anwar Jusuf

**Affiliations:** 1 Department of Medical Education, Faculty of Medicine, Universitas Pelita Harapan, Karawaci, Indonesia; 2 Department of Medical Education, Faculty of Medicine, Universitas Indonesia, Jakarta, Indonesia

**Keywords:** Autonomous motivation, autonomy support, academic achievement, self-determination theory

## Abstract

**Objectives:**

This study applied self-determination theory (SDT) to investigate the relationship between students’ autonomous motivation and tutors’ autonomy support in medical students’ academic achievement.

**Methods:**

This was a cross-sectional study. Out of 204 students in a fundamental medical science course, 199 participated in the study. Data was collected using two questionnaires: the Learning Self-Regulation and Learning Climate Questionnaires. The score of the course assessment was the measure of academic achievement. Data was analyzed and reported with descriptive and inferential statistics (mean, standard deviation and multiple regression analysis).

**Results:**

Mean score (±standard deviation) of the autonomous motivation, tutors’ autonomy support, and academic achievement were 5.48±0.89, 5.22±0.92, and 5.22±0.92. Multiple regression results reported students’ autonomous motivation was associated with improvement of students’ academic achievement (β=15.2, p=0.004). However, augmentation of tutors’ autonomy support was not reflected in the improvement of students’ academic achievement (β = -12.6, p = 0.019). Both students’ autonomous motivation and tutors’ autonomy support had a contribution of about 4.2% students’ academic achievement (F = 4.343, p = 0.014, R^2^ = 0.042).

**Conclusions:**

Due to the unique characteristic of our medical students’ educational background, our study shows that tutors’ autonomy support is inconsistent with students’ academic achievement. However, both autonomous motivation and support are essential to students’ academic achievement. Further study is needed to explore students’ educational background and self-regulated learning competence to improve students’ academic achievement.

## Introduction

There is a transformation of motivation theory in both medical and non-medical fields that is leaning toward qualitative instead of quantitative theory. The limitation of quantitative theory is the inability to explain different levels of motivation in students, such as why some students are highly motivated while others have low motivation. Self-determination theory (SDT) is a qualitative theory that can explain different levels of motivation. The theory is gaining popularity in medical education because it provides new insights that high motivation can be achieved through teachers’ autonomy support and learning environment.^1^^-^^3^ Although the amount of SDT application and research in medical education is limited, some results have successfully demonstrated that high self-determination motivation plays an important role in increasing medical students’ academic achievement, learning strategies, study efforts and reducing students’ exhaustion during the learning process.^4^^-^^6^  The high quality of self-determination motivation can be obtained with three basic psychological needs of the students, which are satisfied through learning environment supports. The three basic psychological needs are autonomy, competence and relatedness.^1^ These supports will improve medical students’ academic achievements, communication skills and retention during teaching and learning activities.^7^^, ^^8^

Studies have proved that SDT is an important variable to increase students’ academic achievement. However, the scale of its association to academic achievement is still unknown.^4^^-^^6^ Some studies for SDT application in medical education to assess the direct relationship between high self-determination motivation or autonomous motivation and teachers’ autonomy support in students’ academic achievement are still rare^3^^,^^4^ and the learning environment setting used in those studies is still very unspecific.^4^^,^^5^^,^^7^

### Self-determination theory

The SDT was introduced by Deci and Ryan, which has recently become very popular for the ability to clarify different types of motivation based on qualities of which other motivation theories are incapable of clarifying.^9^  SDT is influenced by two basic theories: (a) Cognitive evaluation theory: students’ motivation can be facilitated or diminished during the students’ learning process and learning environment, and (b) Organismic integration theory: fulfillment of students’ basic psychological needs is the key to continuing the process of internalization and students will continue to experience the feeling of autonomy even though their behaviors are influenced by external factors, such as regulations set by teachers or institutions.^1^^,^^4^^,^^7^

Based on the aforementioned basic theories, the spectrum of motivation according to SDT can be divided into four major categories: (a) human behavior, (b) type of motivation, (c) type of regulation, and (d) locus of causality or the origin of motivation.10 In terms of human behavior, the qualities of students’ motivation are measured from low to high determination behavior. The lowest determination behavior is a motivation type (students with lack of motivation during their learning process). Then, the quality of determination behavior gradually increases to the right, from extrinsic to intrinsic motivation. That change can be seen from the factors affecting students to participate actively during the tutorial learning process. At the beginning, they are motivated because of external factors, such as a teachers’ pressure or reward; in time, their motivation changes into an intrinsic one, such as their own interest, pleasure or satisfaction of basic psychological needs. Intrinsic motivation is the highest determination behavior.^1^^,^^3^^,^^11^^,^^12^

According to the type of regulation, extrinsic motivation (EM) can be divided into four categories. First, EM with external regulation is usually characterized with students’ behavior that is influenced by external factors and the main reason for obeying the rules is to avoid punishment. Second, EM with introjected regulation refers to the students’ behavior which is majorly influenced by external factors, such as to attain respect from others, to stop feeling guilty, or to avoid social and peer groups’ rejection. Third, EM with identified regulation refers to students’ behavior as influenced by real understanding of the rules. The existing rules have undergone a partial process of internalization into the students’ value. Fourth, EM with integrated regulation refers to students’ behavior as the result of the assimilation of identified regulation into students’ value. In reality, integrated regulation cannot be distinguished by intrinsic motivation (IM) and the instrument for measuring this type of motivation is still undiscovered.^1^^,^^3^^,^^4^

Moreover, EM with external and introjected regulations can be classified as controlled motivation, in which the origin of motivation comes from external factors. While, EM with identified regulation, integrated regulation and intrinsic motivation are classified as autonomous motivation, in which the origin of motivation comes from internal factors and the fulfilment of students’ basic psychological needs.^1^ According to SDT, motivation is a process and continuum; students’ EM can develop to IM or vice versa; and it is then known as the term relative autonomous motivation (RAM). RAM can be defined as how much students’ motivation originated from themselves (autonomous) and can be measured by subtracting the score of students’ controlled motivation from autonomous motivation.^4^

According to SDT, in a social context, the relationship between teachers and students significantly contributes to the transformation of autonomous motivation to controlled motivation or vice versa during the learning process. This depends on how much autonomy support is granted from teachers to the students. However, teachers are recommended to give autonomy support and structure concurrently for novice students who have poor self-regulated learning skills.^9^^,^^13^^,^^14^ Teachers enforce structure in place to help students engage in learning and become competent.^13^ The structure is defined as teachers’ advice to their students on students’ behavior. For example, for students with poor self-regulated learning skills, teachers are advised to provide help in the forms of communicating clear goals or expectation, self-regulated learning strategies and regularly evaluating students’ progression. The structure will improve students’ self-regulated learning skills and autonomy support will encourage students to stay consistent during the study.^13^^,^^15^

Based on previous studies, a cross-sectional observational study was designed with a problem-based learning (PBL) discussion as the research setting.  The purpose of the study was to investigate how students’ autonomous motivation and tutors’ autonomy support were associated with medical students’ academic achievement with teacher-centered educational background and poor self-regulated learning skills. Three research questions navigated this study: (a) How is the relationship between students’ autonomous motivation and their academic achievement? (b) How is the relationship between tutors’ autonomy support and students’ academic achievement? and (c) How is the relationship between both students’ autonomous motivation and tutors’ autonomy support in students’ academic achievement?

## Methods

### Study design and setting

A cross-sectional study was conducted at UPH medical school, one of the private medical schools in Indonesia. The study was performed on first-year students enrolled in a fundamental medical science (FMS) course, from January to March 2016.

### Participants

This study used total sampling scheme. Out of 204, 199 (97.50%) participated in the study by responding to the questionnaires and taking a course assessment.  All participants were between the ages of 17-40, and 61.30% were female (n = 122). Before participating in the discussion for the FMS course, these students were trained for six weeks in a beginner PBL course during their first semester.

### Data collection method

Data was collected using two types of SDT questionnaires: Learning Self-Regulation Questionnaire (LSRQ) and Learning Climate Questionnaire (LCQ). LSRQ is a 12-item questionnaire to measure students’ motivation. It consists of two sub-scales: autonomous motivation (7-item; α = 0.80) and controlled motivation (5-item, α = 0.75). Participants indicate their disagreement or agreement with each statement on a 7-point Likert scale from 1 (not true at all) to 7 (very true). The sub-scale score was obtained by averaging the total score from the items.^16^

Learning Climate Questionnaire is a 15-item questionnaire to measure students’ perception towards their tutors during PBL discussion in FMS. Participants indicate their disagreement or agreement with each statement on a 7-point Likert scale from  1 (strongly disagree) to 7 (strongly agree) with Cronbach alpha ranging from 0.93 to 0.94.^17^ Since the language instruction at UPH medical school is in English, then both LSRQ and LCQ do not need to be translated. A pilot study was conducted before data collection to validate the content of both questionnaires. A 3-point scale item content validity index (I-CVI) was done to rate the relevancy, clarity, simplicity and ambiguity. I-CVI value of > 0.78 was considered a good content validity item.^18^ Thirty students from 2014 class were randomly selected to participate in the pilot study.^19^ Three and five statements of LSRQ and LCQ, respectively, were revised after the study to avoid ambiguity by clarifying and increasing the relevancy of the statements to conform with the PBL discussion. For example, the original LCQ2 stated: “I feel understood by my tutor”, then it was revised to: “My tutor understands my strengths and weaknesses during PBL discussion sessions”. The revised questionnaires were then distributed to all participants in this study.

Students’ academic achievement was measured using a 100-item multiple choice test. This test is a regular summative test in the course. The items in this test were dichotomously scored and with Cronbach alpha of 0.87.^20^

### Procedure

A packet with the questionnaires and informed consent was distributed to participants in the third week of their FMS course. The informed consent explained the purpose of the study and the nature of the study with voluntary participation.  The participants were asked to complete the informed consent before data collection. Participants had 10 minutes to complete the questionnaires. Meanwhile, the students’ academic achievement data was obtained from UPH medical school assessment team at the end of the course. This study was conducted with the approval of the Ethics Committee of the Faculty of Medicine, University of Indonesia and UPH Dean, Faculty of Medicine.

### Data analysis

Data collected was checked for completeness and accuracy. Using SPSS (version 11.5), the obtained data was analyzed by using means, standard deviation and multiple linear regression analysis. Multiple linear regression analysis was conducted to examine the relationship and the influence of students’ autonomous motivation and tutors’ autonomy support towards students’ academic achievement.^21^

## Results

The two sub-scales of the LSRQ (autonomous motivation and controlled motivation) had an average mean (±standard deviation) of 5.48±0.89 and 4.70±0.97. The average of the LCQ for tutor autonomy support was 5.22±0.92. The average of multiple choice tests for academic achievement was 64.70±11.13. More than half of the respondents (79.40%) were autonomously motivated. Both students’ autonomous support and perception towards tutors’ autonomy support (Appendix 1) were high (5.48 and 5.22 out of 7). The Cronbach alpha of different scales ranged from 0.75 to 0.93. 

### Multiple regression modelling for prediction of academic achievement

Multiple linear regression analysis was performed using autonomous motivation and tutors’ autonomy support as independent variables and the score of the regular summative test in FMS course as the outcome variable. Four principal assumptions were used to justify a linear regression model in this study to predict students’ academic achievement: normality, heteroscedasticity, multicollinearity, and autocorrelation tests. The tests revealed that the regression model in this study had a normal distribution value, free from heteroscedasticity, no correlation within independent variables and without autocorrelation (Table 1).

**Table 1:** Principal assumptions tests for regression model diagnostics

Principal Assumptions Tests		Residual	Significant 2-tailed (Spearman’s rho)	Tolerance	VIF	DW
Normality (Kolmogorov-Smirnov)		0.900*				
Heteroscedasticity						
	Students’ autonomous motivation		0.442*			
	Tutors’ autonomy support for students		0.161*			
Multicollinearity						
	Students’ autonomous motivation			0.559^†^	1.790^‡^	
	Tutors’ autonomy support for students			0.559^†^	1.790^‡^	
Autocorrelation						1.823^¶^

*p>0.05 residual value normally distributed and had no heteroscedasticity;

†Tolerance value > 0.1;

‡VIF (variance inflation factor)

### Relationship between students’ autonomous motivation and students’ academic achievement

Partial coefficient regression analysis (t value) shows that autonomous motivation was significantly associated with students’ academic achievement. An increase of 1% students’ autonomous motivation was associated with 15.2% improved academic achievement (p=0.004, β=15.2). This illustration is only employed to students’ with zero percent of tutors’ autonomy support.

### Relationship between tutors’ autonomy support and students’ academic achievement      

In contrast to autonomous motivation, tutors’ autonomous support was negatively associated with students’ academic achievement. An increase of 1% tutors’ autonomy support was associated with 12.6% decreased students’ academic achievement (p=0.019, β=12.6). This association could only be considered if there was zero percent of students’ autonomous motivation.

### Relationship between students’ autonomous motivation and tutors’ autonomy support in students’ academic achievement

There was a significant relationship between students’ autonomous motivation and tutors’ autonomy support in students’ academic achievement (p=0.014, F=4.343). Both students’ autonomous motivation and tutors’ autonomy support gave 4.2% influence on students’ academic achievement (R^2^=0.042). The remaining 95.8% students’ academic achievement was influenced by other variables which were not included in this multiple regression model. Summary of the multiple regression analysis can be seen in Table 2.

**Table 2:** The relationship between autonomous motivation and tutors’ autonomy support with students’ academic achievement

Independent Variable	Unstandardized Coefficients β	t Test		F Test		R^2^
		t	p-value^*^	F	p-value^*^	
Constant	59.669	8.045	0.000			
Autonomous motivation	15.206	2.876	0.004			
Tutors’ autonomy support	-12.600	-2.360	0.019			
Autonomous motivation and tutors’ autonomy support				4.343	0.014	0.042

*p<0.05 indicates that independent variable is partially and simultaneously associated with dependent variable (academic achievement).

## Discussion

The current study was the first one that applied SDT to establish the direct relationship between autonomous motivation and tutors’ autonomy support in Indonesian medical students’ academic achievement in PBL discussion setting.

As the results of the study indicate, students who had high autonomous motivation tended to gain high academic achievement, suggesting that high-quality motivation would likely contribute to students’ learning desire. Strong learning desire improves students’ academic achievement.^1^^,^^7^ The first finding may indicate that autonomous motivation was necessary for improving students’ academic achievement.

The finding above seems to be consistent with Williams and Deci’s longitudinal study. Students with high autonomous motivation had better biopsychosocial value towards patients’ health care and their interpersonal relationship.^8^ However, contradictory results were reported in other studies, such as Black and Deci did not find the direct association of autonomous motivation on improving students’ academic achievement.^7^ In contrast, they found that an upsurge in autonomous motivation tended to improve students’ academic achievement in organic chemistry training. Sobral^5^ and Kusurkar et al.^4^ studies showed that autonomous motivation improved medical students’ academic achievement indirectly through deep approach learning strategies and dedication to self-directed learning. The result of the study shows tutors’ autonomy support tended to diminish students’ academic achievement. This finding seems to be inconsistent with other researchers. These researchers found that the teachers’ autonomy support were essential in developing students’ future competence, clinical interviewing skills and academic achievement.^7^^,^^8^^,^^22^

There are several possible explanations as to why the tutors’ autonomy support resulted in the diminished students’ academic achievement in this study. The main reason might be attributable to the students’ educational background. Their educational background was still predominantly influenced by a teacher-centered environment, and students were not well-equipped with self-regulated learning skills. Changes in teaching and learning methods from teacher-centered to student-centered environments in Indonesia occurred in 2013 after the regulation issued by the Ministry of Education and Culture of the Republic of Indonesia.^23^ Related to this issue, there is a possibility the participants were not accustomed to the student-centered teaching and learning method. According to Dunbar in Bernardus’ study, the culture of teacher-centered methods had been attached to Indonesian students; therefore, it is not easy to change this character. Teacher-centered educational background has caused students to be unprepared for self-regulated learning when tutors provided autonomy support.^24^

Second, the medical students had poor self-directed learning readiness (SDLR). Based on previous studies in Indonesia depicted that about 32.9% - 64% first year medical and nursing students had below average level of SDLR scores. This condition might be connected with low- level self-regulated learning skills, for example, the inability of students to determine which part should be studied, knowing the best learning method, managing study time and taking responsibility for themselves.^24^^, ^^25^

Based on SDT studies, the medical students could be considered as novice students with a lack of self-regulated learning skills. As their confidence in practicing self-regulated learning skills grows, their academic achievement can be influenced by tutors’ autonomy support in a positive way. Hence, to overcome this situation, tutors need to provide structure and autonomy support concurrently as advised in SDT.^9^^,^^13^^-^^15^

Students’ self-regulated learning skills can be improved through three components of the structure. First, tutors ask the students to read course books from the institution, in particular discussing the expected goal and competence which should be achieved after the discussion ended. Second, tutors can provide suggestions and supervision for students with low self-regulated learning skills. The last component is the positive and constructive feedback given by tutors following a discussion.^13^^,^^14^

The result of the study also demonstrates an effective contribution of both students’ autonomous motivation and tutors’ autonomy support towards students’ academic achievement. This finding indicates that students’ autonomous motivation and tutors’ autonomy support may be important determinants in developing students’ academic achievement. This finding is supported by previous SDT studies.  According to SDT, a high level of tutors’ autonomy support to students will promote a higher autonomous motivation level thus improving students’ academic achievement.^1^ Another study by Black and Deci found that tutors played a role in developing students’ autonomous motivation as well as students’ academic achievement.^7^

### Limitations and strengths

The strength of this study was the employment of multiple linear regression analysis to assess the relationship between students’ autonomy motivation and tutors’ autonomy support in medical students’ academic achievement. Through this analysis, an effective contribution scale of these two determinants of academic achievement can be calculated. Moreover, this study was also able to show similar results with longitudinal studies, namely the association of autonomous motivation with academic achievement. This may result from the fact that the participants in this study had been trained in PBL methods during their first six weeks of the medical course corresponding to suggestions from Black and Deci.^7^ To the authors’ knowledge, this was the first study that investigated the direct relationship between autonomous motivation and tutors’ autonomy support in Indonesian medical students’ academic achievement in PBL discussion setting. Therefore, the results of this study could be used as a reference for future studies of SDT application in other medical education institutions in Indonesia.

There are several limitations inherent in this study. First, the questionnaires were administered to students who had been trained in PBL methods before. Therefore, the social desirability bias is clear, and we were not able to control it.  Second, this study did not investigate the reasons for low contribution values of both autonomous motivation and tutors’ autonomy support on students’ academic achievement. Third, though there are possibilities of other unknown variables, two independent variables (students’ autonomous motivation and tutors’ autonomy support) were correlated with one dependent variable (students’ academic achievement) in this study. Other unknown variables are students’ perceptions of learning environments, intelligence level, self-regulated learning skills, and structures.

### Implication for research, medical institutions and medical educators

Previous studies establish the positive relation of tutors’ autonomy support with students’ academic achievement.^7^^,^^8^ This study may serve as a starting point for further research on the impact of high tutors’ autonomy support given to students’ with the teacher-centered educational background.  This finding has offered an additional perspective to the medical education literature that tutors’ autonomy support associated negatively with students’ academic achievement, especially students with the teacher-centered educational background and poor self-regulated learning skills. 

The findings suggest implications for medical institutions to train their medical educators on how to apply SDT during the teaching and learning process. Medical educators as teachers are encouraged to give autonomy support as well as structure, especially for students with low self-regulated learning skills. Moreover, medical educators are expected to apply the SDT behavior from the training process to their daily life. Proper implementation of SDT during the teaching and learning process will satisfy the students’ basic psychological needs. The satisfaction of basic psychological needs will maintain a good quality of students’ autonomous motivation and facilitate a motivational change from the lowest to the highest one (intrinsic motivation). High motivation has a major impact on increasing students’ academic achievement.

## Conclusions

Three significant findings are resulted in this study on students’ autonomous motivation, tutors’ autonomous motivation and academic achievement. The study has established a direct association between students’ autonomous motivation and academic achievement. Conversely, the tutors’ autonomy support has an inverse association with students’ academic achievement. Both students’ autonomous motivation and tutors’ autonomy support, contribute a significant impact on students’ academic achievement. Based on the findings, exploring the students’ educational background and self-regulated learning competence are suggested before SDT implementation. This exploration will have potential benefits to guide medical educators to provide proper SDT behavior for the improvement of students’ academic achievement.

Furthermore, qualitative research needs to be included in the future studies to investigate factors that cause low academic achievement for students having the high autonomous motivation and high tutors’ autonomy support. For longitudinal study, it is recommended to take a sampling from more than one year class. It is also suggested to add more variables in the future study to investigate the independency of each variable or factor affecting students’ academic achievement.

### Acknowledgements

We would like to thank Winta Indra Agus, Austin Ryan Church and Lynn Jones as the editors of this article; and medical students who participated in this research.  

### Conflict of Interest

The authors declare that they have no conflict of interest.

## Supplementary material

Supplementary file 1 Appendix 1. Students’ perception towards tutors’ autonomy support
