# Supplementary material for: The relationship between autonomous motivation and autonomy support in medical students’ academic achievement
Source: Int J Med Educ. 2016 Dec 29;7:417–23. doi: 10.5116/ijme.5843.1097 (PMC5203800; doi:10.5116/ijme.5843.1097)
Supplement: Supplementary file 1 — Appendix 1. Students’ perception towards tutors’ autonomy support [file ijme-7-417-S1.pdf]

Appendix 1

Students' perception towards tutors' autonomy support

Code	Instrument of Learning Climate Questionnaire (LCQ) - N=199<br>Tutors' Autonomy Support	Mean
LCQ6	My tutor made sure I really understood the goals of the course and what I need to do.	5.49
LCQ11	My tutor manages students' emotions appropriately during problem-based learning tutorial sessions.	5.43
LCQ9	My tutor answers my questions fully and carefully.	5.42
LCQ7	My tutor encouraged me to ask questions.	5.38
LCQ8	I feel a lot of trust in my tutor.	5.38
LCQ13	I don't feel very good about the way my tutor talks to me.*	5.33*
LCQ10	My tutor listens to how I would like to do things.	5.29
LCQ12	I feel that my tutor cares about me as a person.	5.26
LCQ3	I am able to be open with my tutor during class.	5.25
LCQ1	I feel that my tutor provides me choices and options in learning objectives and looking for references.	5.20
LCQ4	My tutor conveyed confidence in my ability to do well in the course.	5.18
LCQ2	My tutor understands my strengths and weaknesses during problem-based learning tutorial sessions.	5.04
LCQ14	My tutor tries to understand how I see things before suggesting a new way to do things.	5.03
LCQ5	I feel that my tutor acknowledge my personality as a whole.	4.90
LCQ15	I am able to communicate my feelings with my tutor.	4.72

*This is a negative intonation sentence. The scores are reversed for measurement purposes.
